# Comment on the prevalence of oral frailty among older adults: a systematic review and meta‑analysis

**DOI:** 10.1007/s41999-024-00991-2

**Published:** 2024-05-20

**Authors:** Jin-feng Zhang, Cheng Liu, Zaigui Luo, Xiaomin Xie, Weibo Wei, Yunzhi Yang, Xiaoming Zhang

**Affiliations:** 1 Emergency Department, The People’s Hospital of Baoan Shenzhen, Shenzhen, China; 2 Guangxi University of Chinese Medicine, Guangxi, China; 3 Nursing Department, The People’s Hospital of Baoan Shenzhen, Shenzhen, China

Dear Editor,

We recently read the article titled “The prevalence of oral frailty among older adults: a systematic review and meta‑analysis” conducted by Li and colleagues [1]. Oral frailty is a new concept in the field of geriatrics, which has garnered significant attention from researchers worldwide. It is defined as poor oral function in older people, including declined chewing, swallowing difficulties, poor oral motor skills, and issues with tongue pressure. Tanaka [2] first proposed oral frailty and letter other researcher developed several assessments for it, such as the Oral Frailty Index-8, Oral Frailty checklist, and Oral Frailty Index-6. Li and their colleagues summarized the pooled prevalence of oral frailty among older people, finding it to be 24% (95% CI 15.8–28%), and the prevalence of oral pre-frailty to be 57% (95% CI 52–61%). They also noted higher prevalence rates of oral frailty among females, in hospital settings, in China, and in cross-sectional studies compared to their counterparts. This systematic review contributes significantly to drawing attention to oral health in older populations, aiding dentists and geriatricians in focusing on this important aspect of healthcare. However, there are some aspects that need discussion to improve the quality of the meta-analysis.

First, based on the search strategy and selection criteria, we believe that some important articles may have been unintentionally omitted. For example, Tanaka’s study [3], “Oral Frailty Five-Item Checklist to Predict Adverse Health Outcomes in Community-Dwelling Older Adults,” explored the association between oral frailty and adverse outcomes and reported a baseline prevalence of oral frailty of 39.3%, which we suggest should be included in this meta-analysis. Additionally, we have identified five other important articles that should be included (see supplemental Table 1). Therefore, we have re-summarized the included articles and re-analyzed the pooled prevalence of oral frailty. The results show a prevalence of 23% (95% CI 18–28%) across 24 studies, which was slightly lower than Li's study, showed in supplemental Fig. 1.

Second, there are numerous assessment tools for confirming oral frailty, such as the Oral Frailty Index-8, Oral Frailty Index-6, and Oral Frailty Checklist. Although these tools may have some common items, there are also differences in specific items among them. Therefore, we suggest that the authors provide detailed information on each item and the cutoff points for assessing oral frailty. This information will help readers better understand why there are different prevalence rates of oral frailty when conducting subgroup analyses based on diagnostic criteria.

Third, while the authors conducted subgroup analyses based on different variables such as gender, source, study design, region, and scale for oral frailty, providing p-values for the differences in each stratum would confirm whether there are significant differences in prevalence among subgroups. For example, subgroup analysis based on settings found a prevalence of oral frailty of 23.3% (95% CI 17.6–29.5%) in community settings and 30.8% (95% CI 17.4–46.1%) in hospital settings, with a p-value of 0.53, indicating no significant difference. Similarly, subgroup analysis based on study design showed a prevalence of oral frailty of 25.7% (95% CI 19.2–32.9%) in cross-sectional studies and 21.3% (95% CI 13.5–30.4%) in cohort studies, with no significant difference (*P* = 0.42). Additionally, subgroup analysis based on diagnostic criteria found the highest prevalence of oral frailty with the OFI-8 (43.50%, 95% CI 33.5–53.8%), followed by OFI-6 (17.1%, 95% CI 14.7–19.7%), and others (28.0%, 95% CI 9.9–50.9%), with a significant difference (*p* < 0.01). The subgroup analysis based on country showed that the prevalence of oral frailty was 30.3% (95%CI 17.1–45.5%) for China, 23.3% (95%CI 17.42–29.8%) for Japan, and 17.7% (95%CI 13.9–21.9%) for Finland.

In summary, Li and colleagues have done an excellent job following the PRISMA principles. They conducted comprehensive statistical analyses, including subgroup analyses, sensitivity analysis, meta-regression, and assessment of publication bias. Most importantly, they provided a valuable summary of the current evidence on the overall prevalence of oral frailty among older people. Given the increasing importance of oral health in older populations, understanding the epidemiology of oral frailty is crucial for dentistry and geriatrics, enabling medical professionals and researchers to contribute more effectively to improving oral health. We hope our suggestions can help to improve the quality of this meta-analysis regarding for this important issue.

### Supplementary Information

Below is the link to the electronic supplementary material.

Supplementary file1 (PDF 12 KB)

Supplementary file2 (DOC 126 KB)
