# Supplementary figures and images for: Comment on the prevalence of oral frailty among older adults: a systematic review and meta‑analysis
Source: Eur Geriatr Med. 2024 May 20;15(3):871–2. doi: 10.1007/s41999-024-00991-2 (PMC11329386; doi:10.1007/s41999-024-00991-2)

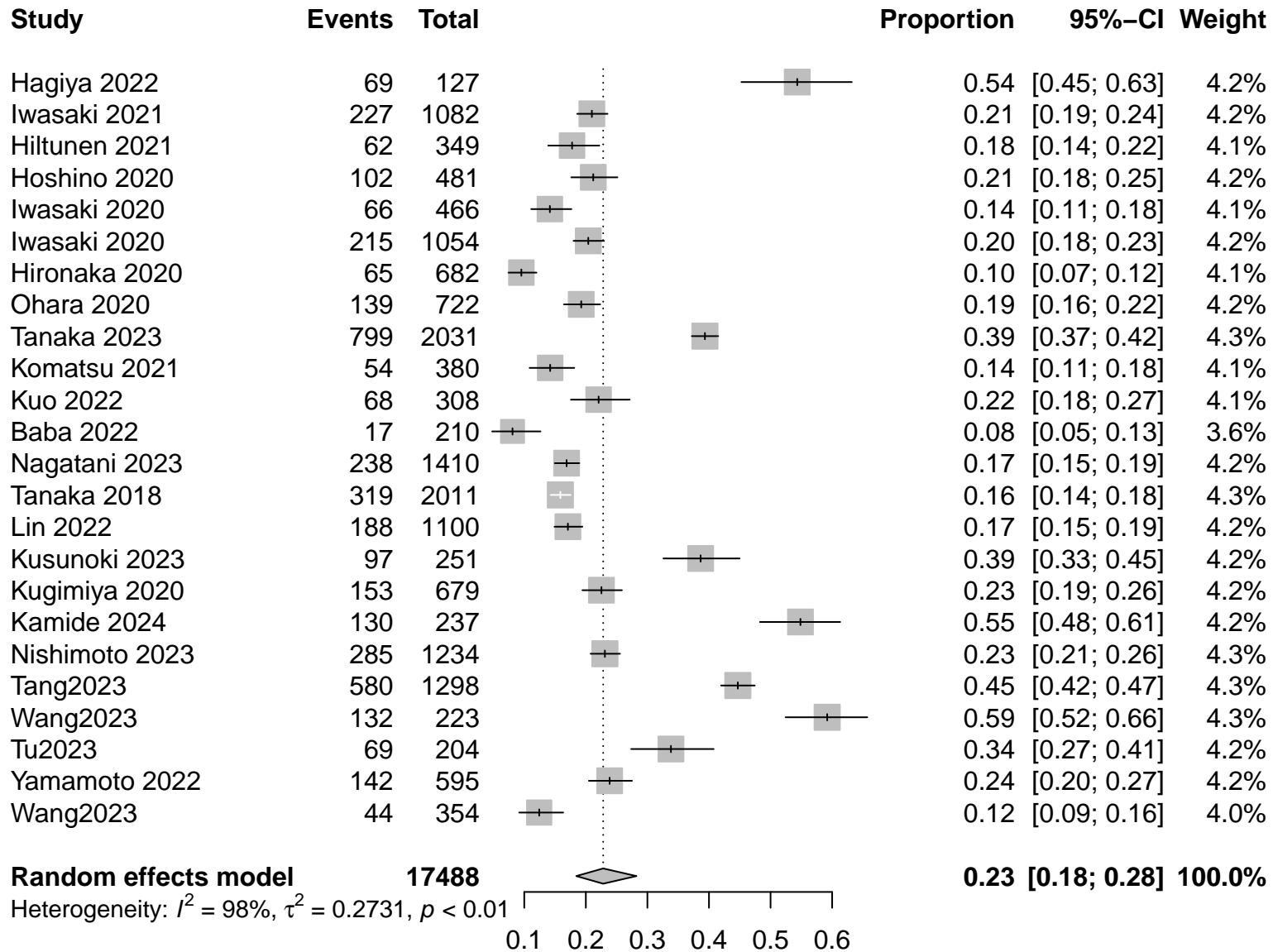

Figure1: The meta-analysis for the prevalence of oral frailty among older people

Supplement: Supplementary file 1 — Supplementary file1 (PDF 12 KB) [file 41999_2024_991_MOESM1_ESM.pdf]
