# Supplementary material for: Comment on the prevalence of oral frailty among older adults: a systematic review and meta‑analysis
Source: Eur Geriatr Med. 2024 May 20;15(3):871–2. doi: 10.1007/s41999-024-00991-2 (PMC11329386; doi:10.1007/s41999-024-00991-2)
Supplement: Supplementary file 2 — Supplementary file2 (DOC 126 KB) [file 41999_2024_991_MOESM2_ESM.doc]

Supplemental Table1: Characteristics of included studies

| author/year | Country | study design | Setting | mean age/medium | Sample size | diagnostic | Number of oral frailty | Prevalence of oral frailty |
| --- | --- | --- | --- | --- | --- | --- | --- | --- |
| Hagiya 2022^[1]^ | Japan | Cross-sectional | Others | 73 | 127 | OFI-8 | 69 | 54.33% |
| Iwasaki 2021^[2]^ | Japan | Cross-sectional | Community | 77.1 | 1082 | OFI-6 | 227 | 20.98% |
| Hiltunen 2021^[3]^ | Finland | Cross-sectional | Others | 82 | 349 | others | 62 | 17.77% |
| Hoshino 2020^[4]^ | Japan | Cross-sectional | Community | 75.9 | 481 | OFI-6 | 102 | 21.21% |
| Iwasaki 2020^[5]^ | Japan | Cohort study | Community | 76.4 | 466 | OFI-6 | 66 | 14.16% |
| Iwasaki 2020^[6]^ | Japan | Cross-sectional | Community | 77 | 1054 | OFI-6 | 215 | 20.40% |
| Kuo 2022^[7]^ | China | Cross-sectional | Community | 79.7 | 308 | OFI-8 | 186 | 60.39% |
| Hironaka 2020^[8]^ | Japan | Cross-sectional | Community | 73.3 | 682 | OFI-6 | 65 | 9.53% |
| Ohara 2020^[9]^ | Japan | Cross-sectional | Community | 79.1 | 722 | OFI-6 | 139 | 19.25% |
| Tanaka 2023^[10]^ | Japan | Cohort study | Community | 73.1 | 2031 | others | 799 | 39.09% |
| Nishimoto 2023^[11]^ | Japan | Cohort study | Community | 72.2 | 1234 | OFI-6 | 285 | 23.10% |
| Komatsu 2021^[12]^ | Japan | Cross-sectional | Community | 72.8 | 380 | OFI-6 | 54 | 14.21% |
| Baba 2022^[13]^ | Japan | Cross-sectional | Community | 74.2 | 210 | OFI-6 | 17 | 8.10% |
| Nagatani 2023^[14]^ | Japan | Cohort study | Community | 72.4 | 1410 | OFI-6 | 238 | 16.88% |
| Tanaka 2018^[15]^ | Japan | Cohort study | Community | 73 | 2011 | OFI-6 | 319 | 15.86% |
| Lin 2022^[16]^ | China | Cross-sectional | Community | None | 1100 | OFI-6 | 188 | 17.09% |
| Kusunoki 2023^[17]^ | Japan | Cross-sectional | Hospital | 77.7 | 251 | OFI-8 | 97 | 38.65% |
| Kugimiya 2020^[18]^ | Japan | Cross-sectional | Community | 75 | 679 | OFI-6 | 153 | 22.53% |
| Kamide2024^[19]^ | Japan | Cross-sectional | Community | 76 | 237 | OFI-8 | 130 | 54.85% |
| Tang2023^[21]^ | China | Cross-sectional | Community | 72.7 | 1298 | OFI-8 | 580 | 44.68% |
| Wang2023^[22]^ | China | Cross-sectional | Community | none | 223 | OFI-8 | 132 | 59.19% |
| Tu2023^[23]^ | China | Cross-sectional | Community | 72.71 | 204 | OFI-8 | 69 | 33.82% |
| Yamamoto ^[20]^2022 | Japan | Cross-sectional | Hospital | NR | 595 | OFI-6 | 142 | 23.87% |
| Wang2023^[24]^ | China | Cross-sectional | Community | 71.6 | 354 | OFI-6 | 44 | 12.43% |

[1] HAGIYA H, TAKASE R, HONDA H, et al. Prevalence of medical factors related to aging among older car drivers: a multicenter, cross-sectional, descriptive study [J]. BMC geriatrics, 2022, 22(1): 792.

[2] IWASAKI M, WATANABE Y, MOTOKAWA K, et al. Oral frailty and gait performance in community-dwelling older adults: findings from the Takashimadaira study [J]. Journal of prosthodontic research, 2021, 65(4): 467-73.

[3] HILTUNEN K, SAARELA R K T, KAUTIAINEN H, et al. Relationship between Fried's frailty phenotype and oral frailty in long-term care residents [J]. Age and ageing, 2021, 50(6): 2133-9.

[4] HOSHINO D, HIRANO H, EDAHIRO A, et al. Association between Oral Frailty and Dietary Variety among Community-Dwelling Older Persons: A Cross-Sectional Study [J]. The journal of nutrition, health & aging, 2021, 25(3): 361-8.

[5] IWASAKI M, MOTOKAWA K, WATANABE Y, et al. A Two-Year Longitudinal Study of the Association between Oral Frailty and Deteriorating Nutritional Status among Community-Dwelling Older Adults [J]. International journal of environmental research and public health, 2020, 18(1).

[6] IWASAKI M, MOTOKAWA K, WATANABE Y, et al. Association between Oral Frailty and Nutritional Status among Community-Dwelling Older Adults: the Takashimadaira Study [J]. The journal of nutrition, health & aging, 2020, 24(9): 1003-10.

[7] KUO Y W, LEE J D. Association between Oral Frailty and Physical Frailty among Rural Middle-Old Community-Dwelling People with Cognitive Decline in Taiwan: A Cross-Sectional Study [J]. International journal of environmental research and public health, 2022, 19(5).

[8] HIRONAKA S, KUGIMIYA Y, WATANABE Y, et al. Association between oral, social, and physical frailty in community-dwelling older adults [J]. Archives of gerontology and geriatrics, 2020, 89: 104105.

[9] OHARA Y, MOTOKAWA K, WATANABE Y, et al. Association of eating alone with oral frailty among community-dwelling older adults in Japan [J]. Archives of gerontology and geriatrics, 2020, 87: 104014.

[10] TANAKA T, HIRANO H, IKEBE K, et al. Oral frailty five-item checklist to predict adverse health outcomes in community-dwelling older adults: A Kashiwa cohort study [J]. Geriatrics & gerontology international, 2023, 23(9): 651-9.

[11] NISHIMOTO M, TANAKA T, HIRANO H, et al. Severe Periodontitis Increases the Risk of Oral Frailty: A Six-Year Follow-Up Study from Kashiwa Cohort Study [J]. Geriatrics (Basel, Switzerland), 2023, 8(1).

[12] KOMATSU R, NAGAI K, HASEGAWA Y, et al. Association between Physical Frailty Subdomains and Oral Frailty in Community-Dwelling Older Adults [J]. International journal of environmental research and public health, 2021, 18(6).

[13] BABA H, WATANABE Y, MIURA K, et al. Oral frailty and carriage of oral Candida in community-dwelling older adults (Check-up to discover Health with Energy for senior Residents in Iwamizawa; CHEER Iwamizawa) [J]. Gerodontology, 2022, 39(1): 49-58.

[14] NAGATANI M, TANAKA T, SON B K, et al. Oral frailty as a risk factor for mild cognitive impairment in community-dwelling older adults: Kashiwa study [J]. Experimental gerontology, 2023, 172: 112075.

[15] TANAKA T, TAKAHASHI K, HIRANO H, et al. Oral Frailty as a Risk Factor for Physical Frailty and Mortality in Community-Dwelling Elderly [J]. The journals of gerontology Series A, Biological sciences and medical sciences, 2018, 73(12): 1661-7.

[16] LIN Y C, HUANG S S, YEN C W, et al. Physical Frailty and Oral Frailty Associated with Late-Life Depression in Community-Dwelling Older Adults [J]. Journal of personalized medicine, 2022, 12(3).

[17] KUSUNOKI H, EKAWA K, KATO N, et al. Association between oral frailty and cystatin C-related indices-A questionnaire (OFI-8) study in general internal medicine practice [J]. PloS one, 2023, 18(4): e0283803.

[18] KUGIMIYA Y, WATANABE Y, UEDA T, et al. Rate of oral frailty and oral hypofunction in rural community-dwelling older Japanese individuals [J]. Gerodontology, 2020, 37(4): 342-52.

[19] KAMIDE N, ANDO M, MURAKAMI T, et al. The association of oral frailty with fall risk in community-dwelling older adults: a cross-sectional, observational study [J]. European geriatric medicine, 2024, 15(1): 279-83.

[20] YAMAMOTO T, TANAKA T, HIRANO H, et al. Model to Predict Oral Frailty Based on a Questionnaire: A Cross-Sectional Study [J]. International journal of environmental research and public health, 2022, 19(20).

[24] Wang XY (2021) Oral frailty of the community elderly: the asso‑ciation between sarcopenia and nutrition and depression [D]. Tai‑pei University of Nursing

and Health Sciences
